# Enhanced oxygen consumption in *Herbaspirillum seropedicae fnr* mutants leads to increased NifA mediated transcriptional activation

**DOI:** 10.1186/s12866-015-0432-6

**Published:** 2015-05-07

**Authors:** Marcelo Bueno Batista, Roseli Wassem, Fábio de Oliveira Pedrosa, Emanuel Maltempi de Souza, Ray Dixon, Rose Adele Monteiro

**Affiliations:** Department of Biochemistry and Molecular Biology, Universidade Federal do Paraná, P.O. Box 19046, Curitiba, PR 81531-990 Brazil; Department of Molecular Microbiology, John Innes Centre, Colney Lane, Norwich, NR4 7UH UK; Department of Genetics, Universidade Federal do Paraná, P.O. Box 19071, Curitiba, PR 81531-990 Brazil

**Keywords:** *Herbaspirilum seropedicae*, NifA, Fnr

## Abstract

**Background:**

Orthologous proteins of the Crp/Fnr family have been previously implicated in controlling expression and/or activity of the NifA transcriptional activator in some diazotrophs. This study aimed to address the role of three Fnr-like proteins from *H. seropedicae* SmR1 in controlling NifA activity and consequent NifA-mediated transcription activation.

**Results:**

The activity of NifA-dependent transcriptional fusions (*nifA::lacZ* and *nifB::lacZ*) was analysed in a series of *H. seropedicae fnr* deletion mutant backgrounds. We found that combined deletions in both the *fnr1* and *fnr3* genes lead to higher expression of both the *nifA* and *nifB* genes and also an increased level of *nifH* transcripts. Expression profiles of *nifB* under different oxygen concentrations, together with oxygen consumption measurements suggest that the triple *fnr* mutant has higher respiratory activity when compared to the wild type, which we believe to be responsible for greater stability of the oxygen sensitive NifA protein. This conclusion was further substantiated by measuring the levels of NifA protein and its activity in *fnr* deletion strains in comparison with the wild-type.

**Conclusions:**

Fnr proteins are indirectly involved in controlling the activity of NifA in *H. seropedicae,* probably as a consequence of their influence on respiratory activity in relation to oxygen availability. Additionally we can suggest that there is some redundancy in the physiological function of the three Fnr paralogs in this organism, since altered respiration and effects on NifA activity are only observed in deletion strains lacking both *fnr1* and *fnr3*.

**Electronic supplementary material:**

The online version of this article (doi:10.1186/s12866-015-0432-6) contains supplementary material, which is available to authorized users.

## Background

The endophytic diazotroph *Herbaspirillum seropedicae* SmR1 is a Beta-proteobacterium found in association with economically important crops such as rice, maize, sugar cane and sorghum [1,2]. *H. seropedicae* can fix nitrogen under micro-oxic and nitrogen limiting conditions and expression of *H. seropedicae* nitrogen fixation (*nif*) genes inside plant tissues has been demonstrated [3]. In *H. seropedicae* the *nif* genes are clustered in a contiguous region of 46 genes [4], comprising at least seven operons [1], whose products are essential for biosynthesis, maturation and assembly of the nitrogenase complex. The nitrogenase structural genes (*nifHDK*) are located in the *nifHDKENXHsero2847Hsero_2846fdxA* operon. *nifH* encodes the iron (Fe) protein while *nifDK* encodes the molybenum-iron (MoFe) protein.The *nifB* gene, which encodes a protein involved in the synthesis of FeMoco, is located in an operon with other *nif*-related genes. The σ^54^-dependent transcriptional activator, NifA, a member of the bacterial enhancer binding family [5] is a master regulator of *nif* gene expression in *H. seropedicae* SmR1 [1]. Two sites for NifA binding and a consensus binding site for the RNA polymerase σ^54^ holoenzyme were found in the promoters upstream *nifB* and *nifH* [6,7]*.* NifA responds to both fixed nitrogen and oxygen levels, being activated in response to limitation of these resources [8]. Once active, NifA activates transcription from *nif* promoters [9] including *nifB* and *nifH* (reviewed in [1]).

The Fnr protein, is a widespread transcriptional regulator that binds a [4Fe–4S]^2+^ cluster to monitor the oxygen status in the cell [10], and regulates the transcription of genes required for the metabolic switch in response to decreasing oxygen levels [11-13]. Orthologous proteins of the Crp/Fnr family [11,14] have been previously implicated in controlling expression and/or activity of the NifA transcriptional activator in some diazotrophs [14-16]. In *Klebsiella pneumoniae* Fnr influences NifA activity through modulation of the mechanism by which the NifL repressor protein is sequestered to the membrane [15]. In *Bradyrhizobium japonicum* the Fnr-like protein, FixK_1_, negatively controls genes that are subject to NifA activation [16] suggesting that FixK_1_, can repress transcription at NifA-dependent promoters. Another precedent for Fnr involvement in NifA activity was observed by Monteiro and co-workers [17], who showed that the activity of an amino-terminally truncated form of *H. seropedicae* NifA was influenced by Fnr when expressed in an *Escherichia coli fnr-* background.

The *H. seropedicae* genome [4] has three genes encoding for Fnr-like proteins [1] and a role for these Fnr orthologs in controlling the expression of the complete cytochrome *c* branch of the electron transport chain has been demonstrated [18]. In this study we aimed to investigate the potential involvement of *H. seropedicae* Fnr proteins in the expression and activity of NifA and the consequences for transcriptional activation of other *nif* genes.

We found that combined deletions in both the *fnr1* and *fnr3* genes lead to higher expression of *nifB::lacZ* and *nifA::lacZ* transcriptional fusions and increased *nifH* transcription. We also show that the oxygen consumption rate in multiple *fnr* deletion strains is higher than in the wild-type, which we believe to result in either higher stability or activity of the oxygen sensitive NifA protein and consequently increased transcriptional activation of *nif* genes.

## Results and discussion

**Figure 1:**
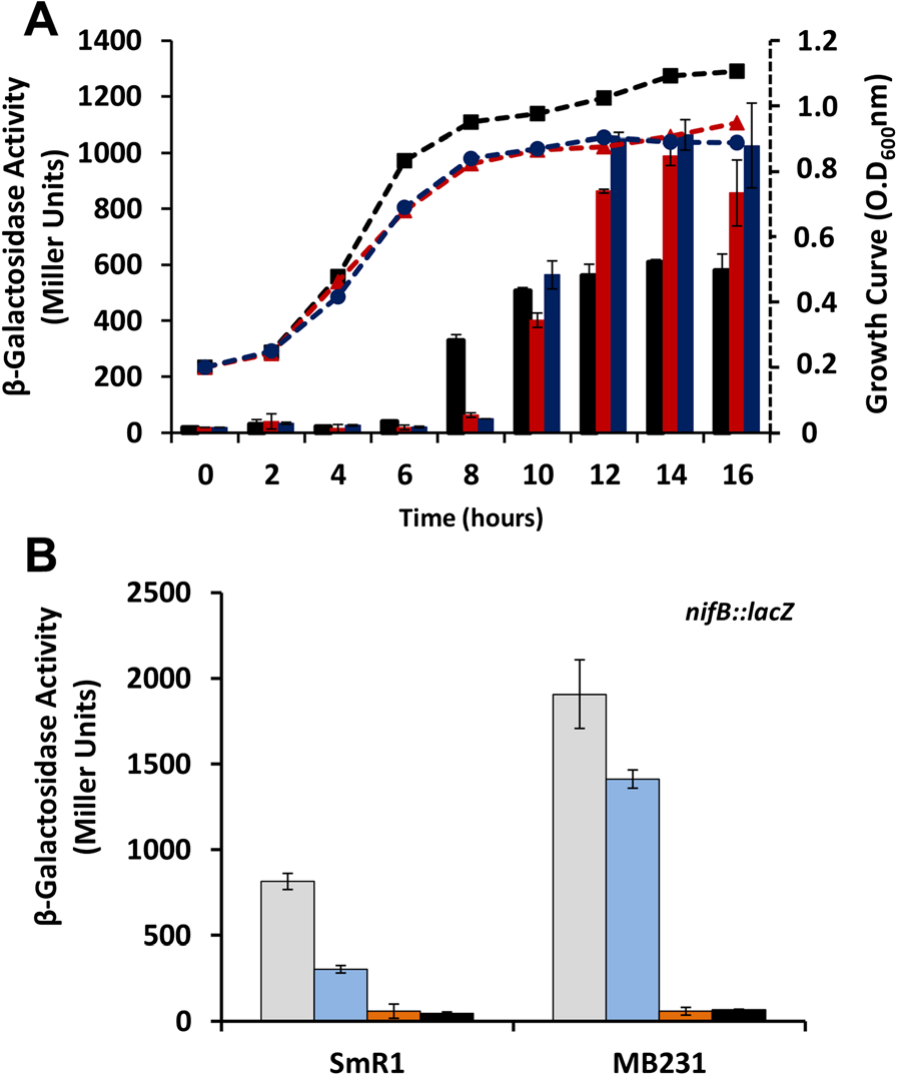
The *nifB* gene expression is enhanced in the *fnr* mutant strains from *H. seropedicae.* **(A)** The activity of the *nifB::lacZ* fusion (primary y axis) was assayed using cells cultured in NFbHP-Malate liquid medium supplemented with 5 mM NH_4_Cl (6 ml in 25 ml bottles under air). Every two hours samples were taken for determination of β-galactosidase activity of the wild type strain (SmR1) (black bars), the double *fnr1* and *fnr3* deletion strain (MB13) (red bars) and the triple *fnr* deletion strain (MB231) (blue bars). Samples were also taken for measuring the growth (O.D_600-nm_ – secondary y axis) of the strains SmR1 (dotted black lines – squares), MB13 (dotted red lines – triangles) and MB231 (dotted blue lines – circles). **(B)** Activity of the *nifB::lacZ* fusion measured under the initial oxygen concentrations of 4.0% (light grey bars), 6.0% (blue bars), 8.0% (orange bars) and 20.8% (black bars) in liquid medium without addition of fixed nitrogen as describe in Methods.

To analyze if the three *fnr* genes in *H. seropedicae* influence either the expression level or the activity of NifA, we monitored expression of a *nifB::lacZ* fusion, as a reporter of NifA activity. We compared *nifB* expression in the wild-type strain (SmR1) with a double deletion strain, which lacks both *fnr1* and *fnr3* (MB13) and a strain carrying deletions in all three *fnr* genes (MB231). Multiple *fnr* deletion strains were not analysed in these experiments since single gene deletions did not influence NifA activity. As *nifB* gene expression is tightly regulated by nitrogen and oxygen levels in the cell [6], the activity of the *nifB::lacZ* reporter fusion is only observed when the cultures exhaust the supply of fixed nitrogen and oxygen becomes limited, as the culture reaches a high cell density. Although the *fnr* deletions impaired growth under these conditions as observed previously [18], the activity of the *nifB::lacZ* fusion was significantly higher after 12–16 hours incubation in the strain lacking both *fnr1* and *fnr3* (MB13) and also in the triple *fnr* deletion strain (MB231) when compared with the wild-type (Figure 1A). This suggests that either NifA expression or its activity is more highly induced in cultures of these multiple *fnr* deletion strains.

**Figure 2:**
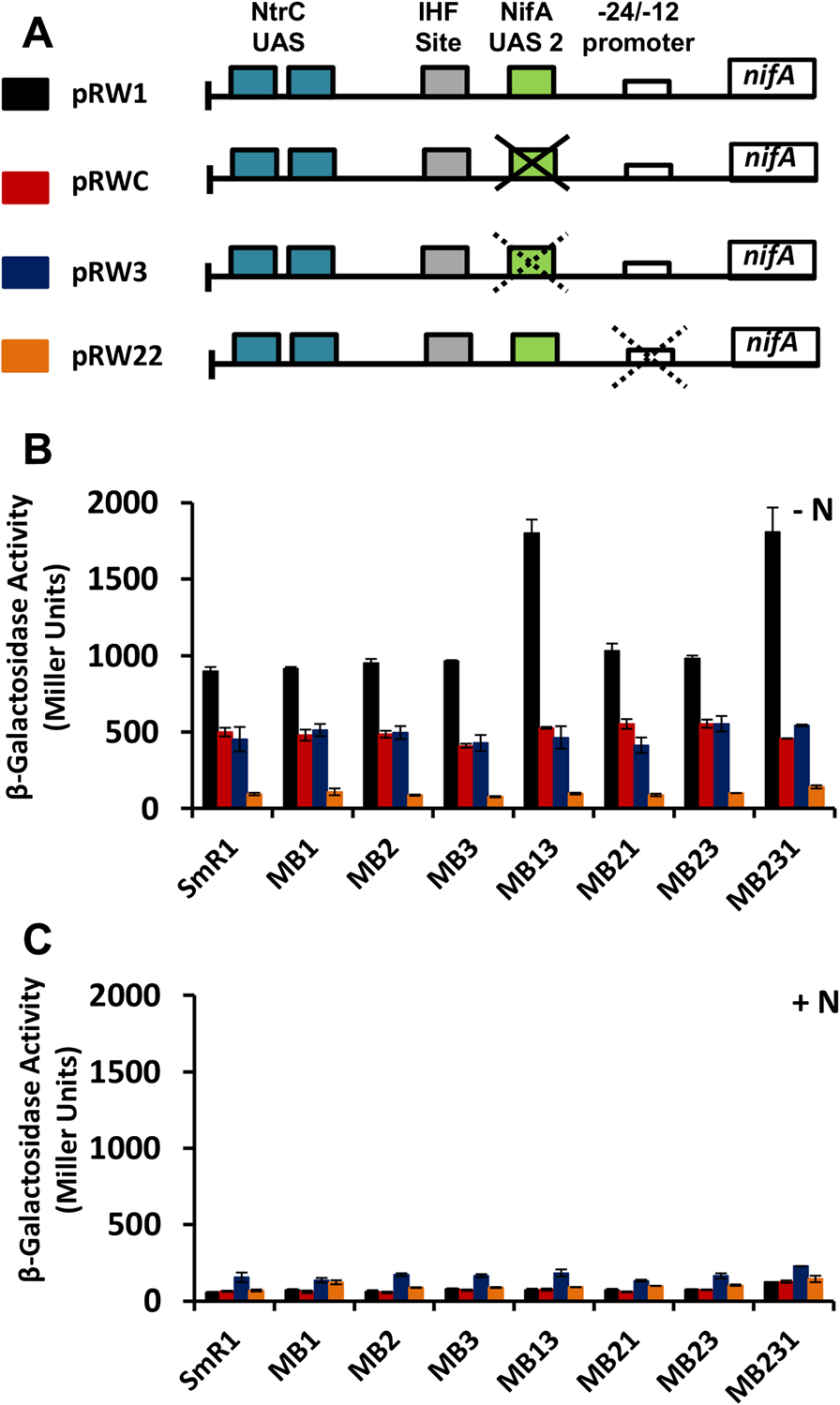
The *nifA* gene expression is enhanced in the *fnr* mutant strains from *H. seropedicae.* **(A)** Schematic representation of native and mutant *nifA::lacZ* fusions assayed for β-Galactosidase activity in *fnr* mutant strains is showed. Full crosses indicate deletions, while dotted crosses indicate point mutations. The β-Galactosidase activity of different *nifA::lacZ* transcriptional fusions in *H. seropedicae* SmR1 and *fnr* mutant strains were assayed under 5 mM **(B)** or 20 mM of NH_4_Cl **(C)**. The colour code used for each transcriptional fusion in the bar graphs in **B** and **C** is represented in **A**. Results showed are representative of two independent experiments.

We considered the possibility that the multiple *fnr* deletion strains exhaust dissolved oxygen in the media faster than the wild type strain, thus leading to higher activity or stability of NifA in cultures of the *fnr* deletion mutants. To examine this further, we assayed *nifB::lacZ* activity in cultures grown in the absence of fixed nitrogen under defined initial oxygen concentrations of oxygen in the gas phase (Figure 1B). As anticipated, *nifB* expression was not detected under either 8% or 20.8% oxygen in both wild-type and the *fnr* triple deletion mutant, presumably because *H. seropedicae* NifA is inactivated at high oxygen concentrations [8,19]. However, the activity of the *nifB::lacZ* promoter fusion was markedly higher in the triple *fnr* deletion strain (MB231) compared with the parental strain, when cultures were incubated under an initial oxygen concentration of 4% or 6% in the gas phase (Figure 1B). To ensure that the increase of *nifB* expression observed in the mutant strains was NifA-dependent, we prepared single *nifA*^*−*^ and multiple deletion strains carrying a *nifA* deletion in addition to the *fnr* mutations (Additional file 1) and confirmed that the influence of Fnr proteins on *nifB* promoter activation requires NifA protein (Additional file 2).

Since expression of the *nifA* gene itself is subject to autoactivation in *H. seropedicae* [20], we tested the influence of *fnr* deletions on *nifA* expression using various *nifA::lacZ* promoter constructs (Figure 2). Transcriptional regulation of *nifA* is complex, since this σ^54^-dependent promoter is subject to nitrogen regulation by the enhancer binding protein NtrC in addition to autogenous activation by NifA under oxygen-limiting conditions (see Figure 2A). Notably, single deletions in each of the three *fnr* genes had no apparent influence on *nifA* expression. However, as in the case of *nifB,* an increase in promoter activation was apparent in the double *fnr1, fnr3* deletion mutant (MB13) and the triple *fnr* deletion strain (MB231) (Figure 2B). In all cases, promoter activation significantly decreased when cultures were grown in the presence of a high concentration of fixed nitrogen (Figure 2C), or when the −24 to −12 region of the promoter was disrupted (plasmid pRW22, Figure 2B), indicating that activation is *rpoN*-dependent and subject to nitrogen regulation by NtrC as expected [20]. In all cases, irrespective of the presence of *fnr* mutations, *nifA* expression decreased when promoter constructs (plasmids pRWC and pRW3) carried mutations in the upstream activation sequence (UAS2) of the promoter (Figure 2B), presumably as a consequence of decreased autoactivation by NifA [20]. Overall, these results demonstrate that in the absence of both *fnr1* and *fnr3*, activation of the *nifA* promoter is increased. Since higher expression of the *nifA::lacZ* fusion is not observed when the NifA binding site (UAS2) is deleted, it is likely that the increased expression results from autoactivation of the *nifA* promoter due to increased activity or stability of NifA protein.

**Figure 3:**
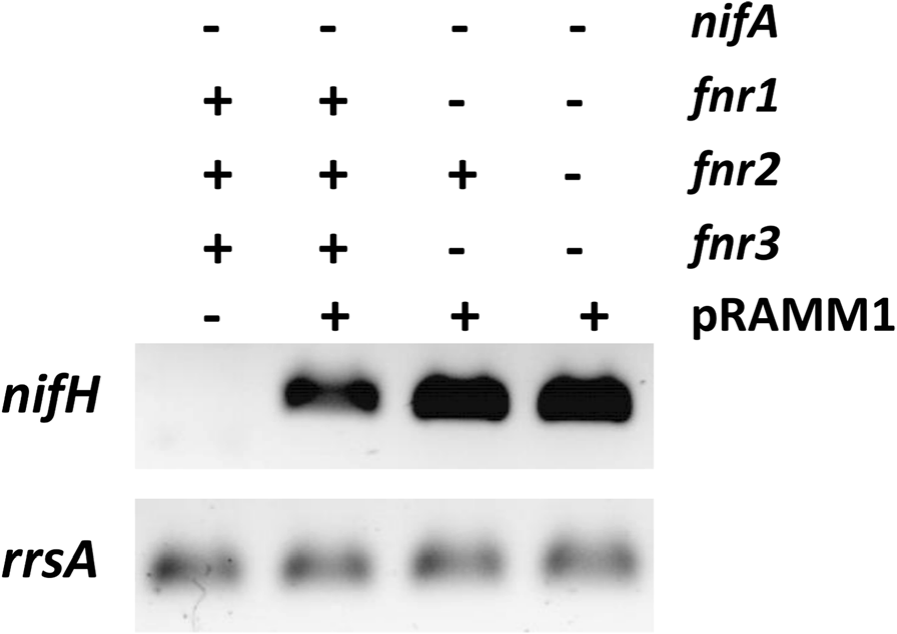
The NifA activity is higher in *H. seropedicae* strains lacking both Fnr1 and Fnr3. The RNA from the strains MBN1 (*nifA* deletion), MBN2 (*nifA* deletion in the double *fnr1, fnr3* deletion background) and MBN3 (*nifA* deletion in the triple *fnr* deletion background) complemented with the plasmid pRAMM1 (expressing *H. seropedicae* NifA from *lac* promoter) was purified and submitted to direct RT-PCR amplification of *nifH* gene as described in Methods. The 16S rRNA (*rrsA*) was used as an endogenous expression control. A representative gel from two independent RNA extractions is showed.

**Figure 4:**
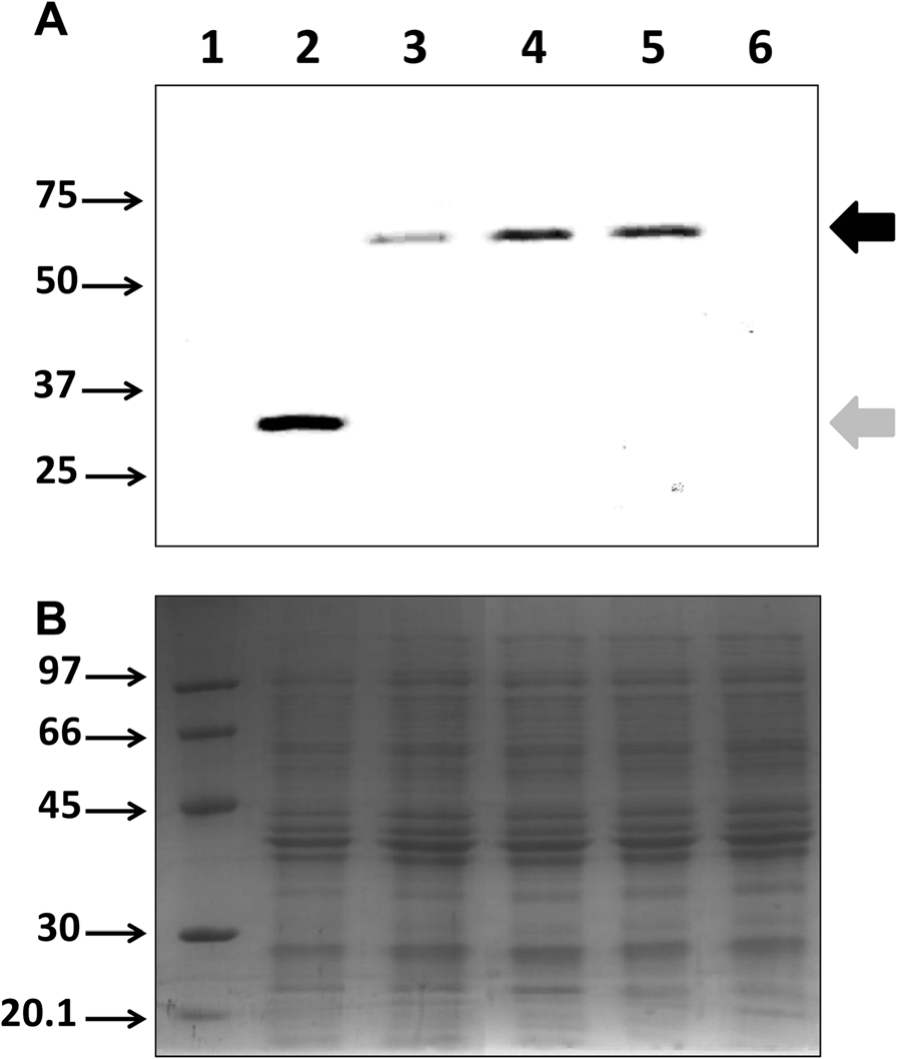
The NifA protein levels are higher in *H. seropedicae* strains lacking both Fnr1 and Fnr3. Protein extracts from different *H. seropedicae* strains were prepared and then hybridized with ANTI-FLAG antibodies as described in Methods. **(A)** Western hybridization of protein samples after resolution on 12% SDS-PAGE and transfer to PVDF membrane. **(B)** Protein loading control SDS-PAGE of samples used for hybridization in A. Lanes are as follows: 1, Protein molecular weight standard (MW); 2, *H. seropedicae* MBF1 (*fnr1-3xFlag*)*;* 3, *H. seropedicae* MBN4 (*nifA*-3xFlag)*;* 4, *H. seropedicae* MBN5 (*nifA-3xFlag* in the double *fnr1*, *fnr3* deletion background)*;* 5, *H. seropedicae* MBN6 (*nifA-3XFlag* in the triple *fnr* mutant background)*;* 6, *H. seropedicae* SmR1 (no Flag protein control). In **A,** the MW used was Precision Plus Protein WesternC™ Pack (BioRad #161-0385), while in **B,** the MW used was the LMW-SDS Marker Kit (GE healthcare# 17-0446-01). Thin arrows indicate the molecular weight in KDa of protein standards. The black thick arrow indicates the NifA-3xFlag protein (~64 KDa), while the light grey thick arrow indicates the Fnr1-3xFlag protein (~34K Da). A representative result is show from three independent protein extract preparations.

Given that the *nifA* promoter is subjected to complex regulation, we designed experiments to confirm that NifA activity is enhanced in *fnr* mutant strains. Firstly, using the combined *fnr*^*−*^ and *nifA* deletion strains described above (Additional files 1 and 2) we complemented the *nifA* mutation with *nifA* expressed ectopically from the *lac* promoter (plasmid pRAMM1), which is constitutive in *H. seropedicae*. In this complementation assay we observed that the levels of *nifH* mRNA were higher in the *fnr* deletion strains complemented with constitutively expressed NifA in comparison with the complemented strain containing wild-type *fnr* alleles (Figure 3). This implies that an increase in NifA activity, rather than its expression, is responsible for increased activation of *nif* promoters in the *fnr* deletion mutants. Secondly, we constructed strains expressing NifA fused to a 3XFlag peptide to allow detection of the protein in both wild type and *fnr* mutant backgrounds (Additional file 3). Western blots of strains carrying the *nifA*-3Xflag allele revealed higher levels of NifA expression in the double *fnr1, fnr3* deletion (MBN5) and also in the triple *fnr* deletion (MBN6) backgrounds compared with the strain carrying wild-type *fnr* alleles (MBN4) (Figure 4). This confirms the additional level of autoactivation of the *nifA* promoter conferred by the multiple *fnr* deletions (Figure 2B), again indicating that NifA activity is higher in the *fnr* mutant strains.

**Figure 5:**
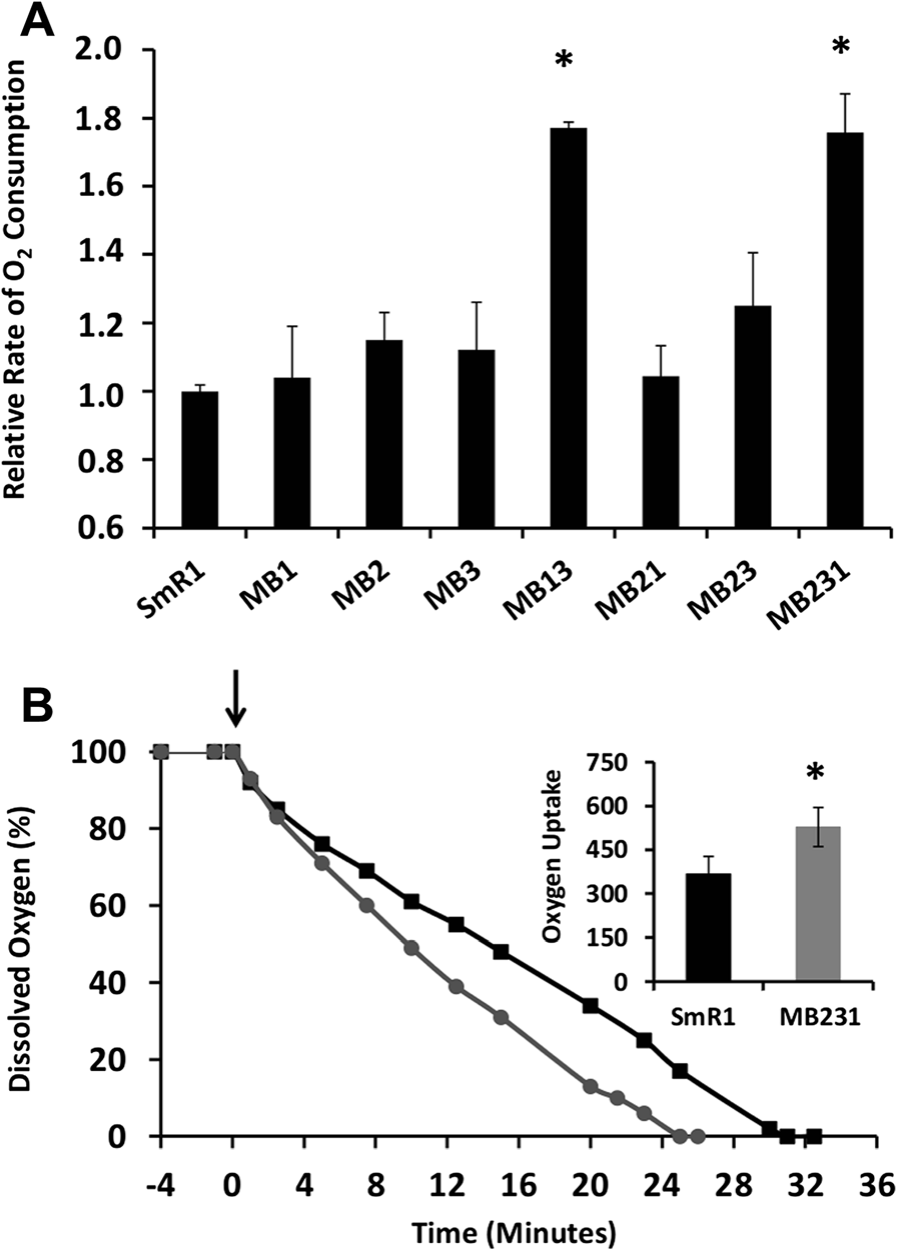
The oxygen consumption rate is higher in the *H. seropedicae fnr* mutant strains. **(A)** Gas phase oxygen consumption in *H. seropedicae* SmR1 and *fnr* mutant strains. **(B)** Consumption of dissolved oxygen in liquid media of *H. seropedicae* strains SmR1 (black squares) and MB231 (grey circles) using a clark-type electrode. The arrow indicates addition of 100 μL of cells into the electrode chamber containing 1.6 ml of NfbHP-Malate supplemented with 5.0 mM of ammonium chloride. The inset shows the specific oxygen uptake rate (given as μmolar O_2_.mg protein.min^−1^) in liquid cultures, which was calculated considering the oxygen solubility in water as 233 μM [25]. The asterisks indicate statistical significance according to the Student’s T-test (P < 0.05), derived from two independent experiments.

As the *H. seropedicae* NifA protein is sensitive to oxygen, being inactivated and degraded upon exposure to O_2_ [8,19], we hypothesized that NifA might be protected in its active form in *fnr* deletion strains if these strains exhibit a higher oxygen consumption rate. To further test this hypothesis we measured oxygen depletion during the growth of bacterial cultures in the same growth conditions as described for the assay of the *nifA::lacZ* fusions. We first analyzed the decrease in oxygen concentration in the gas phase of Suba-seal stoppered flasks (Figure 5A) and additionally compared the profiles of dissolved oxygen consumption using a Clark type electrode (Figure 5B). These assays revealed that the consumption of oxygen was higher in multiple *fnr* deletion strains*,* implying that these strains have a higher respiratory rate when compared to the wild type (Figure 5). Notably, the oxygen consumption data in Figure 5A directly correlates with the increased activity of NifA observed in strains lacking both Fnr1 and Fnr3 (Figure 2B) implying that the absence of both these transcription factors results in higher respiration rates.

In a previous study, we showed that the triple *fnr* mutant is deficient in the expression of the cytochrome *c*-type branch of the electron transport chain [18]. An alternative route of electron transport from the quinol pool to oxygen via the terminal quinol oxidases is likely to occur in the triple *fnr* mutant. As the quinol branch of the respiratory chain results in a lower number of proton-translocation events it is conceivable that the activity of this branch, rather than the expression of the *bo*_*3*_ and *bd*-type oxidases, is enhanced in the *fnr* mutant strains to compensate for the lower level of energy production. This may result in increased electron flux through the respiratory chain and hence enhanced oxygen consumption as observed in our experiments.

We demonstrated previously that nitrogenase activity is severely impaired when the triple *fnr* deletion strain is cultured in ammonium-limiting liquid medium, potentially as a consequence of energy depletion [18]. We also showed that diazotrophic growth is impaired in the *fnr* ablated strain, after subjecting cultures to severe nitrogen starvation [18]. Under these conditions, cultures divide at extremely low growth rates, requiring 24 days post-inoculation to achieve an OD_600_ of ~ 1.6 (Additional file 4). However, it is notable that the triple *fnr* mutant grew faster than the wild-type for the first 12 days of incubation under these conditions. Potentially, the enhanced rate of O_2_ consumption by the triple *fnr* deletion allows higher levels of NifA activity and consequently higher nitrogenase activity during the ‘early’ stages of growth. However, it is possible that as the bacterial population increases and the oxygen levels in the culture drop further, the triple *fnr* mutant strain can no longer maintain the necessary electron flux to support nitrogenase activity and as a consequence, diazotrophic growth is impaired.

In summary, these studies have not identified a direct role for the *H. seropedicae* Fnr proteins in regulating NifA activity and nitrogen fixation, but rather suggest that they may influence both, by means of altering the composition of the electron transport chain and the oxygen consumption rate. Since we only observe such effects in strains deleted for both *fnr1* and *fnr3*, there is apparently some redundancy in the physiological functions of the three *fnr* paralogs in *H. seropedicae.* It is feasible that *H. seropedicae* can take advantage of the three *fnr* genes to differentially modulate respiratory chain composition. This is likely to influence nitrogen fixation during different phases of growth and enable efficient adaptation during plant-bacterial colonization.

## Conclusions

In this study we have used a combination of transcriptional and physiological approaches to address the role of the *H. seropedicae* Fnr proteins in influencing the expression and activity of NifA. In summary we found that Fnr1 and Fnr3 participate indirectly in modulating NifA stability as a consequence of alterations in the rate of O_2_ consumption. This mechanism can potentially allow the bacteria to fine tune nitrogen fixation in response to environmental cues.

## Methods

### Plasmids, bacterial strains and growth conditions

Plasmids and bacterial strains used are listed in Table 1. *H. seropedicae* strains were grown at 30°C in NFbHP-Malate medium [21] supplemented with NH_4_Cl as indicated. The antibiotics used were tetracycline (10 μg mL^−1^), streptomycin (80 μg mL^−1^), kanamycin (500 μg mL^−1^ for *H. seropedicae* and 50 μg mL^−1^ for *E. coli*), gentamicin (500 μg mL^−1^ for *H. seropedicae*) and nalidixic acid (5 μg mL^−1^).

**Table 1:** **Plasmids and strains used in this study**

**Plasmids**	**Relevant characteristic**	**Source**
pLAFR3.18	pLAFR vector containing the polycloning site of pTZ18R, Tc^R^	[8]
pRAMM1	*H.seropedicae* NifA expressed from *lac* promoter	[26]
pK18mobsacBKm	Allelic exchange suicide vector; mobilized by *E. coli* S17-1 λpir, *sacB,*Km^R^	[22]
pJQ200SK	Allelic exchange suicide vector; mobilized by *E. coli* S17-1, *sacB,*Gm^R^	[23]
pRAM1T7	*H.seropedicae* NifA in pT7-7 vector	This study
pRAM1T7del	*H.seropedicae* NifA with a deletion of 576 bp	This study
pK18nifAdel	XbaI/BamHI fragment from pRAM1T7del	This study
pUC57Simple-3xFlag	5′-BamHI, KpnI, XhoI – 3xFlag-Stop – HindIII, SalI, XmaI-3′, Amp^R^	Genscript® Corporation
pUC57nifAFlag	*H. seropedicae nifA-3xFlag* gene plus 647 bp of the downstream region, Amp^R^	This study
pK18nifAFlag	*H. seropedicae nifA-3xFlag* gene plus 647 bp of the downstream region, Mob, SacB, Km^R^	This study
pUC57fnr1Flag	*H. seropedicae fnr1-3xFlag* gene plus 1000 bp of the downstream region, Amp^R^	This study
pJQfnr1Flag	*H. seropedicae fnr1-3xFlag* gene plus 1000 bp of the downstream region, Mob, SacB, Gm^R^	This study
pPW452	*lacZ* fusion vector, Tc^R^, Mob	[27]
pEMS140	Tc^R^, Mob, *nifB::lacZ* fusion, *nifB* promoter cloned in pPW452	[6]
pRW1	Tc^R^, Mob, *nifA::lacZ* fusion, *nifA* promoter cloned in pMP220	[20]
pRWC	pRW1, but with a 49 bp deletion including the UAS 2 site for NifA	[20]
pRW3	pRW1, but with a double mutation at the UAS 2 site for NifA (TGT ->TCT and ACA -> AGA)	[20]
pRW22A	pRW1, but with a single mutation at the −24/-12 promoter (G -> T at −25 residues)	[20]
**Strains**	**Relevant characteristic**	**Source**
SmR1	*Herbaspirillum seropedicae* Z78 but Sm^R^ 100 μg/mL, Nif+	[24]
MB1	Derived from SmR1 *fnr1 deletion*	[18]
MB2	Derived from SmR1, *fnr2* deletion	[18]
MB3	Derived from SmR1, *fnr3* deletion	[18]
MB13	Derived from MB1, *fnr1* and *fnr3* double deletion	[18]
MB21	Derived from MB2, *fnr1* and *fnr1* double deletion	[18]
MB23	Derived from MB2, *fnr2* and *fnr3* double deletion	[18]
MB231	Derived from MB23, *fnr1*, *fnr2* and *fnr3* triple deletion	[18]
MBN1	Derived from SmR1, but with 576 bp deletion in the *nifA* gene	This study
MBN2	Double *fnr1*, *fnr3* deletion, plus a 576 bp deletion in the *nifA* gene	This study
MBN3	Triple *fnr* deletion, plus a 576 bp deletion in the *nifA* gene	This study
MBN4	Derived from SmR1, but with a C-terminal 3xFlag *nifA* gene	This study
MBN5	Double *fnr1*, *fnr3* deletion, plus a C-terminal 3xFlag *nifA* gene	This study
MBN6	Triple *fnr* deletion, plus a C-terminal 3xFlag *nifA* gene	This study
MBF1	Derived from SmR1, but with a C-terminal 3xFlag *fnr1* gene	This study

### Construction of nifA deletion and 3xFlag tagged strains

To construct the C-terminal 3xFlag tagged NifA strains, we generated a *nifA*-3XFlag gene by cloning the complete *nifA* gene (1629 bp) in frame with the 3xFlag sequence from a vector synthesized by the GenScript® Corporation (Table 1). To assist homologous recombination, a fragment of 647 bp downstream of *nifA* was cloned adjacent to the 3xFlag tag sequence to generate an approximately 2.4 Kb fragment containing the *nifA*-3xFlag allele plus the downstream region. This fragment was then digested with BamHI, and subcloned into pK18mobsacBKm vector [22] to generate the suicide vector pK18nifAflag. A similar approach was used to generate a vector for C-terminal 3xFlag tagging of the *fnr1* gene, but a fragment of 1002 bp downstream of *fnr1* gene was cloned adjacent to the 3xFlag tag sequence to generate a fragment of approximately 1.95 kb containing the *fnr1*-3xFlag allele plus the downstream region. This fragment was then subcloned into pJQ200SK suicide vector [23] to generate pJQfnr1Flag. To generate the *nifA* deletion vector, plasmid pRAM1T7 was digested with EcoRI and re-ligated to yield the vector pRAM1T7del containing a deleted copy of *nifA* lacking 576 bp. Then an XbaI/BamHI fragment from pRAM1T7del was cloned into pK18mobsacB vector to generate the pK18nifAdel suicide vector.

The suicide plasmids generated for both tagging of *nifA* and *fnr1* and also for deletion of *nifA* gene were transferred to wild type *H. seropedicae* SmR1, and the *fnr* deletion strains MB13 and MB231 strains by conjugation as described [18,24]. Single crossover strains were selected by antibiotic resistance. Double crossover strains were selected on plates containing 5% sucrose and then tested for antibiotic marker sensitivity. The mutant strains sensitive to kanamycin or gentamicin and resistant to sucrose were analysed by PCR using specific primers as described (Additional files 1, 3 and 5). All primers used are listed in the Additional file 6.

### β-Galactosidase activity and transcriptional fusions

β-Galactosidase activities of various *nif promoter:lacZ* transcriptional fusions were assayed in *H. seropedicae* strains as previously described [6,20], except that the strains were grown in NFbHP-Malate liquid medium supplemented with 5 mM NH_4_Cl (6 ml in 25 ml cylindrical bottles under air). Under these conditions, the cultures exhaust the supply of fixed nitrogen and become oxygen limited resulting in formation of active NifA and *nif* gene expression.

Alternatively, *H. seropedicae* strains carrying the *nifB::lacZ* fusion were assayed for β-galactosidase activity after incubation under defined initial oxygen concentrations. In summary, cultures with an O.D_600_ adjusted to 0.2, were incubated for six hours in NFbHP-Malate without addition of fixed nitrogen and under the initial oxygen concentrations of 4%, 6% or air (20.8%).

### RNA extraction and RT-PCR

Strains were grown under 4% of oxygen for six hours. Cells from 30 mL of culture were collected by centrifugation (7000 rpm, 4°C, 5 minutes) and re-suspended in 200 μL of 10 mM Trizma® (Sigma# T-2694). The cells were then mixed with 700 μL of RLT Buffer (Qiagen Rneasy Mini Kit #74104) containing 1% of β-mercaptoethanol and added to lysing tubes containing zirconia and silica/glass beads in the proportion of 2:1 (Thistle Scientific Ltd). Lysis was carried out with 3 pulses (speed 6.5 with 30 seconds on/1.5 minutes off) using the Thermo Savant FastPrep 120 Cell Disrupter System. Beads and cellular debris were collected by centrifugation (17000 × *g*, 4°C, 5 min). The supernatant (900 μL) was transferred to a new RNase free tube and 450 μL of ethanol (Sigma #459844) was then added. The samples were applied to the RNeasy columns (Qiagen RNeasy Mini Kit #74104) and total RNA was recovered after *on column* DNAse treatment with the Qiagen RNase-Free DNase set (#79254) following the manufacturer’s instructions. The quality of purified RNA was accessed by electrophoresis in a 1% agarose gel. RNA was treated with Turbo DNase (Ambion#AM1907) following manufacturer’s instructions and further purified with Qiagen RNeasy columns to avoid carryover of divalent cations.

Approximately 0.25 ng/μL of total RNA was used for direct RT-PCR using the One-Step RT-PCR kit (Qiagen #210210) according to the manufacturer’s instructions. Expression of *nifH* gene was evaluated using 16S rRNA as endogenous control. The primers are listed in the Additional file 6.

### Preparation of protein extracts and western blotting

*H. seropedicae* cultures adjusted to an O.D_600_ of 0.2 were grown under 4% of oxygen for six hours. After incubation, approximately 3 mL of cells (volumes were adjusted as necessary) were collected by centrifugation (17000 × *g*, 2 min), re-suspended in 100 μL of protein sample buffer (120 mM of Tris–HCl pH 6.8, 2% SDS, 20% Glycerol, 9% β-mercaptoethanol and 0.03% bromophenol blue) and boiled for 5 minutes. Subsequently, 10 μL of the resulting extract was loaded onto 12% SDS-PAGE for resolution of the proteins, which were immediately transferred to a PVDF membrane and then hybridized with ANTI-FLAG® (Sigma #7425) primary antibody (1/2500 dilution), followed by secondary anti-rabbit-HRP conjugated antibody (1/10000 dilution). The HRP activity was detected using the ECL Plus Western Blotting detection kit (GE Healthcare #RPN2132) as indicated by the manufacturer and the UVP® gel imaging system.

### Oxygen consumption measurements

For determination of the oxygen consumption we designed two assays. First we evaluated the depletion of the oxygen levels in the gas phase of culture flasks sealed with Suba-seal septa. Every hour we took a 0.5 ml gas sample from the growing culture and analyzed the oxygen concentration by gas chromatography (Varian GC-450) coupled to a molecular sieve column and a TCD detector. Oxygen depletion was linear until 10 hours growth. The rate of consumption was calculated as the amount of oxygen consumed in the gas phase normalized by the protein concentration of the culture. A measurement of the dissolved oxygen consumption was also carried out with a Clark-type electrode. After addition of 100 μl of bacterial culture into the chamber, containing 1.6 ml of NFbHP-Malate at 30°C, the consumption of dissolved oxygen in the medium was recorded until the polarizing voltage reached 0 (i.e. 0% oxygen saturation).

## Additional files

Additional file 1:
**Construction and validation of** ***nifA*** **deletion strains in different** ***H. seropedicae*** **backgrounds.** (A) Schematic representation of the *nifA* deletion construct and primers (dotted arrows) designed to validate the mutants. Drawings are not to scale. (B) Genotypic validation of strains MBN1 (*nifA* deletion), MBN2 (*nifA* deletion in the double *fnr1* and *fnr3* deletion background) and MBN3 (*nifA* deletion in the triple *fnr* deletion background). PCR was performed by using primers flanking the region of deletion (as indicated in A). Lanes: 1, 1 Kb ladder Fermentas; 2, SmR1 (Wild type *nifA*); 3 suicide vector (pK18nifAdel);4, intermediate strain for SmR1 background; 5, 6, and 7, final *nifA* deleted strains for SmR1, MB13 and MB231 backgrounds, respectively; 8, no template control using primers to *fnr1* gene; 9,10 and 11, SmR1 genotyping of *fnr1, fnr2* and *fnr3* genes; 12, no template control using primers to *fnr2* gene; 13, 14 and 15, MB13 genotyping of *fnr1, fnr2* and *fnr3* genes; 16, no template control using primers to *fnr3* gene; 17, 18 and 19, MB231 genotyping of *fnr1, fnr2* and *fnr3* genes. On the left are indicated the length in base pairs (bp) of the DNA ladder. Additional file 2:
**The enhanced** ***nifB::lacZ*** **promoter activity in strains lacking both Fnr1 and Fnr3 is dependent upon NifA protein.** The *H.seropedicae* strains, SmR1 (wild type), MB13 (*fnr1* and *fnr3* deletion), MB231 (triple *fnr* deletion), MBN1 (*nifA* deletion), MBN2 (*nifA* deletion in the *fnr1, fnr3* deletion background) and MBN3 (*nifA* deletion in the triple *fnr* deletion background) harbouring the plasmid pEMS140 (*nifB::lacZ*) were assayed for β- Galactosidase activity under nitrogen deficient media and 4.0% (red bars) or 20.8% (blue bars) of oxygen as described in Methods. Additional file 3:
**Construction and validation of** ***nifA-*** **3xFlag strains in different** ***H. seropedicae*** **backgrounds.** (A) Schematic representation of C-terminally 3xFlag tagged construct and primers (dotted arrows) designed to validate the mutants. Drawings are not to scale. (B) Genotypic validation of strains MBN4 (*nifA*-3xFlag), MBN5 (*nifA*-3xFlag in the double *fnr1* and *fnr3* deletion background) and MBN6 (*nifA*-3xFlag in the triple *fnr* mutant background). PCR was performed by using primers flanking the C-terminal region around the insertion of the 3xFlag (as indicated in *A*). Lanes: 1, 1 Kb ladder Fermentas; 2, no template control; 3, SmR1; 4, suicide vector (pK18nifAFlag); 5, 6 and 7, intermediate strains for SmR1, MB13 and MB231 backgrounds; 8,9 and 10, final 3xFlag tagged strains for SmR1, MB13 and MB231 backgrounds. The *fnr* genotypes on different *nifA*-3XFlag backgrounds were verified as showed on Additional file 1. On the left are indicated the length in base pairs (bp) of the DNA ladder. Additional file 4:
**Deletion of** ***fnr*** **genes influences the diazotrophic growth profile.** Both *H. seropedicae* wild type strain (SmR1) (black squares) and the triple *fnr* mutant strain (MB231) (red triangles) were incubated statically at 30°C in NFbHP-Malate minimal media without addition of nitrogen source. Additional file 5:
**Construction and validation of** ***fnr1-*** **3xFlag strain from** ***H. seropedicae.*** (A) Schematic representation of C-terminally 3xFlag tagged construct and primers (dotted arrows) designed to validate the mutant. Drawings are not to scale. (B) Genotypic validation of the strain MBF1 (*fnr1*- 3xFlag). PCR was performed by using primers flanking the C-terminal region around the insertion of the 3xFlag (as indicated in A). Lanes: 1, 1 Kb ladder Fermentas; 2, SmR1; 3, suicide vector (pJQfnr1Flag); 4, intermediate strain and 5 final *fnr1-*3xFlag tagged strain (MBF1). On the left are indicated the length in base pairs (bp) of the DNA ladder. Additional file 6:
**Primers used in this study.**

## References

[1] Chubatsu LS, Monteiro RA, Souza EM, Oliveira MAS, Yates MG, Wassem R, Bonatto AC, Huergo LF, Steffens MBR, Rigo LU, Pedrosa FDO (2012). Nitrogen fixation control in *Herbaspirillum seropedicae*. Plant Soil.

[2] Monteiro RA, Balsanelli E, Wassem R, Marin AM, Brusamarello-Santos LCC, Schmidt MA, Tadra-Sfeir MZ, Pankievicz VCS, Cruz LM, Chubatsu LS, Pedrosa FO, Souza EM (2012). *Herbaspirillum*-plant interactions: microscopical, histological and molecular aspects. Plant Soil.

[3] Roncato-Maccari LDB, Ramos HJO, Pedrosa FO, Alquini Y, Chubatsu LS, Yates MG, Rigo LU, Steffens MBR, Souza EM (2003). Endophytic *Herbaspirillum seropedicae* expresses *nif* genes in gramineous plants. FEMS Microbiol Ecol.

[4] Pedrosa FO, Monteiro RA, Wassem R, Cruz LM, Ayub RA, Colauto NB, Fernandez MA, Fungaro MHP, Grisard EC, Hungria M, Madeira HMF, Nodari RO, Osaku CA, Petzl-Erler ML, Terenzi H, Vieira LGE, Steffens MBR, Weiss VA, Pereira LFP, Almeida MIM, Alves LR, Marin A, Araujo LM, Balsanelli E, Baura VA, Chubatsu LS, Faoro H, Favetti A, Friedermann G, Glienke C (2011). Genome of *Herbaspirillum seropedicae* strain SmR1, a specialized diazotrophic endophyte of tropical grasses. PLoS Genet.

[5] Bush M, Dixon R (2012). The role of bacterial enhancer binding proteins as specialized activators of σ54-dependent transcription. Microbiol Mol Biol Rev.

[6] Rego FGM, Pedrosa FO, Chubatsu LS, Yates MG, Wassem R, Steffens MBR, Rigo LU, Souza EM (2006). The expression of *nifB* gene from *Herbaspirillum seropedicae* is dependent upon the NifA and RpoN proteins. Can J Microbiol.

[7] Machado IM, Yates MG, Machado HB, Souza EM, Pedrosa FO (1996). Cloning and sequencing of the nitrogenase structural genes *nifHDK* of *Herbaspirillum seropedicae*. Braz J Med Biol Res.

[8] Souza EM, Pedrosa FO, Drummond M, Rigo LU, Yates MG (1999). Control of *Herbaspirillum seropedicae* NifA activity by ammonium ions and oxygen. J Bacteriol.

[9] Dixon R, Kahn D (2004). Genetic regulation of biological nitrogen fixation. Nat Rev Microbiol.

[10] Lazazzera BA, Beinert H, Khoroshilova N, Kennedy MC, Kiley PJ (1996). DNA binding and dimerization of the Fe-S-containing FNR protein from *Escherichia coli* are regulated by oxygen. J Biol Chem.

[11] Korner H, Sofia HJ, Zumft WG (2003). Phylogeny of the bacterial superfamily of Crp-Fnr transcription regulators: exploiting the metabolic spectrum by controlling alternative gene programs. FEMS Microbiol Rev.

[12] Spiro S (1994). The FNR family of transcriptional regulators. Antonie Van Leeuwenhoek.

[13] Spiro S, Guest JR (1990). FNR and its role in oxygen-regulated gene expression in Escherichia coli. FEMS Microbiol Lett.

[14] Matsui M, Tomita M, Kanai A (2013). Comprehensive computational analysis of bacterial CRP/FNR superfamily and its target motifs reveals stepwise evolution of transcriptional networks. Genome Biol Evol.

[15] Grabbe R, Klopprogge KAI, Schmitz RA. Fnr is required for NifL-dependent oxygen control of *nif* gene expression in *Klebsiella pneumoniae*. J Bacteriol. 2001;183:1385–1393. 10.1128/JB.183.4.1385-1393.2001 PMC95013 11157952

[16] Mesa S, Hauser F, Friberg M, Malaguti E, Fischer H-M, Hennecke H (2008). Comprehensive assessment of the regulons controlled by the FixLJ-FixK2-FixK1 cascade in *Bradyrhizobium japonicum*. J Bacteriol.

[17] Monteiro RA, Souza EM, Yates MG, Pedrosa FO, Chubatsu LS (2003). Fnr is involved in oxygen control of *Herbaspirillum seropedicae* N-truncated NifA protein activity in Escherichia coli. Appl Environ Microbiol.

[18] Batista MB, Sfeir MZT, Faoro H, Wassem R, Steffens MBR, Pedrosa FO, Souza EM, Dixon R, Monteiro RA (2013). The *Herbaspirillum seropedicae* SmR1 Fnr orthologs controls the cytochrome composition of the electron transport chain. Sci Rep.

[19] Oliveira MAS, Baura VA, Aquino B, Huergo LF, Kadowaki MAS, Chubatsu LS, Souza EM, Dixon R, Pedrosa FO, Wassem R, Monteiro RA (2009). Role of conserved cysteine residues in *Herbaspirillum seropedicae* NifA activity. Res Microbiol.

[20] Wassem R, Pedrosa FO, Yates MG, Rego FGM, Chubatsu LS, Rigo LU, Souza EM (2002). Control of autogenous activation of *Herbaspirillum seropedicae* nifA promoter by the IHF protein. FEMS Microbiol Lett.

[21] Klassen G, Pedrosa FO, Souza EM, Funayama S, Rigo LU (1997). Effect of nitrogen compounds on nitrogenase activity in *Herbaspirillum seropedicae* SMR1. Can J Microbiol.

[22] Schäfer A, Tauch A, Jäger W, Kalinowski J, Thierbach G, Pühler A (1994). Small mobilizable multi-purpose cloning vectors derived from the *Escherichia coli* plasmids pK18 and pK19: selection of defined deletions in the chromosome of *Corynebacterium glutamicum*. Gene.

[23] Quandt J, Hynes MF (1993). Versatile suicide vectors which allow direct selection for gene replacement in Gram-negative bacteria. Gene.

[24] Souza EM, Pedrosa FO, Rigo LU, Machado HB, Yates MG (2000). Expression of the nifA gene of *Herbaspirillum seropedicae*: role of the NtrC and NifA binding sites and of the −24/-12 promoter element. Microbiology.

[25] Estabrook RW (1967). Mitochondrial respiratory control and the polarographic measurement of ADP : O ratios. Methods Enzymol.

[26] Noindorf L, Bonatto AC, Monteiro RA, Souza EM, Rigo LU, Pedrosa FO, Steffens MBR, Chubatsu LS (2011). Role of PII proteins in nitrogen fixation control of *Herbaspirillum seropedicae* strain SmR1. BMC Microbiol.

[27] Woodley P, Buck M, Kennedy C (1996). Identification of sequences important for recognition of *vnf* genes by the VnfA transcriptional activator in *Azotobacter vinelandii*. FEMS Microbiol Lett.

