# Supplementary material for: Enhanced oxygen consumption in Herbaspirillum seropedicae fnr mutants leads to increased NifA mediated transcriptional activation
Source: BMC Microbiol. 2015 May 7;15:95. doi: 10.1186/s12866-015-0432-6 (PMC4422417; doi:10.1186/s12866-015-0432-6)
Supplement: Additional file 1: — Construction and validation of nifA deletion strains in different H. seropedicae backgrounds. (A) Schematic representation of the nifA deletion construct and primers (dotted arrows) designed to validate the mutants. Drawings are not to scale. (B) Genotypic validation of strains MBN1 (nifA deletion), MBN2 (nifA deletion in the double fnr1 and fnr3 deletion background) and MBN3 (nifA deletion in the triple fnr deletion background). PCR was performed by using primers flanking the region of deletion (as indicated in A). Lanes: 1, 1 Kb ladder Fermentas; 2, SmR1 (Wild type nifA); 3 suicide vector (pK18nifAdel);4, intermediate strain for SmR1 background; 5, 6, and 7, final nifA deleted strains for SmR1, MB13 and MB231 backgrounds, respectively; 8, no template control using primers to fnr1 gene; 9,10 and 11, SmR1 genotyping of fnr1, fnr2 and fnr3 genes; 12, no template control using primers to fnr2 gene; 13, 14 and 15, MB13 genotyping of fnr1, fnr2 and fnr3 genes; 16, no template control using primers to fnr3 gene; 17, 18 and 19, MB231 genotyping of fnr1, fnr2 and fnr3 genes. On the left are indicated the length in base pairs (bp) of the DNA ladder. [file 12866_2015_432_MOESM1_ESM.pdf]

Additional file 1.

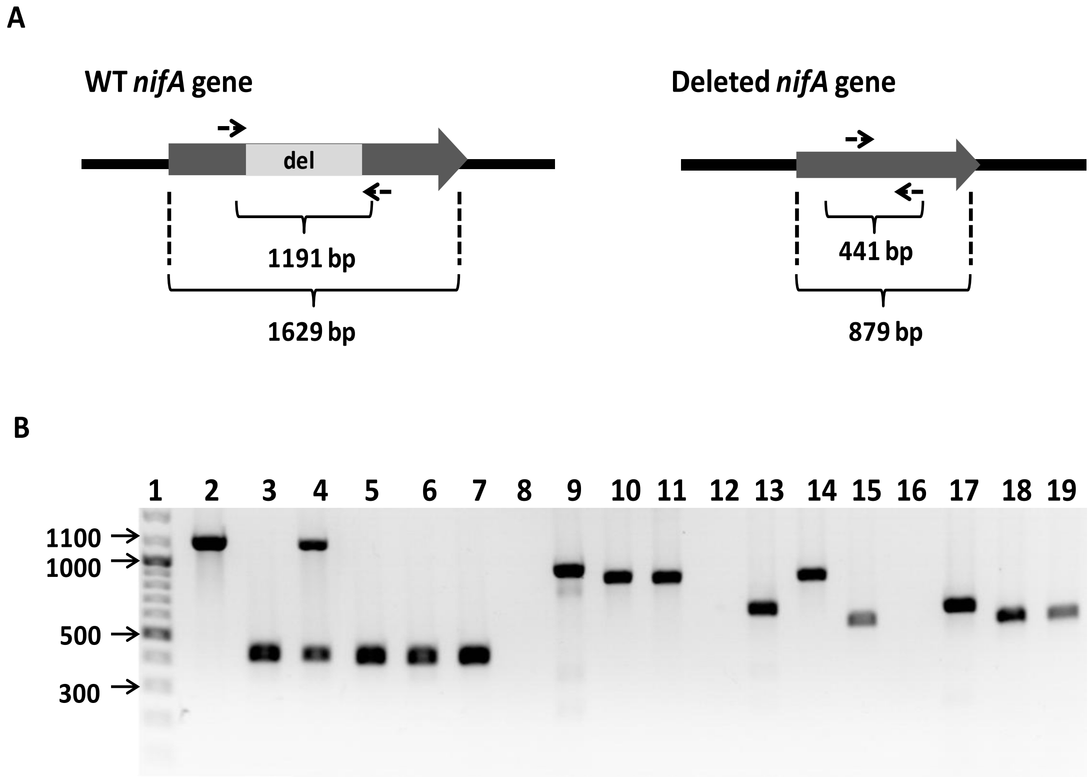

Additional file 1. Construction and validation of *nifA* deletion strains in different *H. seropedicae* backgrounds. (A) Schematic representation of the *nifA* deletion construct and primers (dotted arrows) designed to validate the mutants. Drawings are not to scale. (B) Genotypic validation of strains MBN1 (*nifA* deletion), MBN2 (*nifA* deletion in the double *fnr1* and *fnr3* deletion background) and MBN3 (*nifA* deletion in the triple *fnr* deletion background). PCR was performed by using primers flanking the region of deletion (as indicated in A). Lanes: 1, 1 Kb ladder Fermentas; 2, SmR1 (Wild type *nifA*); 3 suicide vector (pK18nifAdel);4, intermediate strain for SmR1 background; 5, 6, and 7, final *nifA* deleted strains for SmR1, MB13 and MB231 backgrounds, respectively; 8, no template control using primers to *fnr1* gene; 9,10 and 11, SmR1 genotyping of *fnr1, fnr2* and *fnr3* genes; 12, no template control using primers to *fnr2* gene; 13, 14 and 15, MB13 genotyping of *fnr1, fnr2* and *fnr3* genes; 16, no template control using primers to *fnr3* gene; 17, 18 and 19, MB231 genotyping of *fnr1, fnr2* and *fnr3* genes. On the left are indicated the length in base pairs (bp) of the DNA ladder.
