# Supplementary material for: Enhanced oxygen consumption in Herbaspirillum seropedicae fnr mutants leads to increased NifA mediated transcriptional activation
Source: BMC Microbiol. 2015 May 7;15:95. doi: 10.1186/s12866-015-0432-6 (PMC4422417; doi:10.1186/s12866-015-0432-6)
Supplement: Additional file 2: — The enhanced nifB::lacZ promoter activity in strains lacking both Fnr1 and Fnr3 is dependent upon NifA protein. The H.seropedicae strains, SmR1 (wild type), MB13 (fnr1 and fnr3 deletion), MB231 (triple fnr deletion), MBN1 (nifA deletion), MBN2 (nifA deletion in the fnr1, fnr3 deletion background) and MBN3 (nifA deletion in the triple fnr deletion background) harbouring the plasmid pEMS140 (nifB::lacZ) were assayed for β- Galactosidase activity under nitrogen deficient media and 4.0% (red bars) or 20.8% (blue bars) of oxygen as described in Methods. [file 12866_2015_432_MOESM2_ESM.pdf]

Additional file 2.

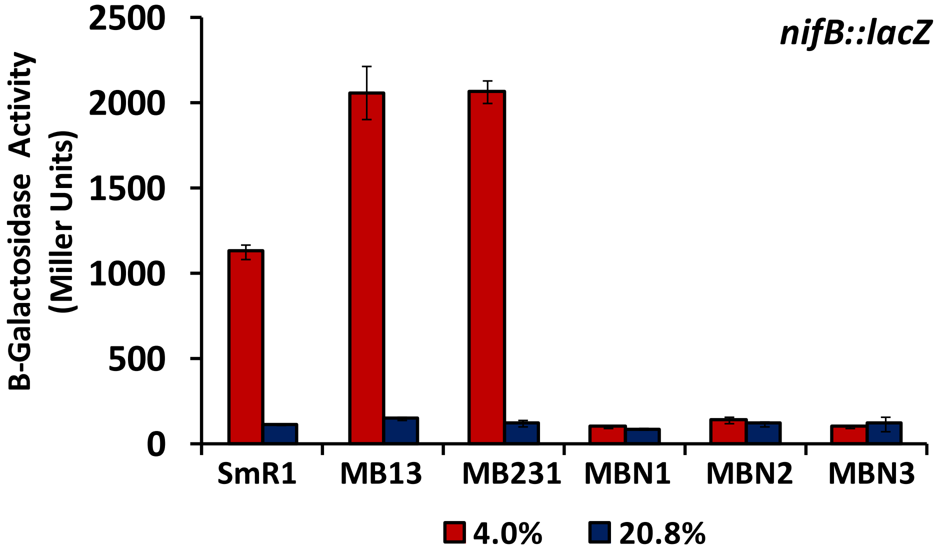

Additional file 2. The enhanced *nifB::lacZ* promoter activity in strains lacking both Fnr1 and Fnr3 is dependent upon NifA protein. The *H.seropedicae* strains, SmR1 (wild type), MB13 (*fnr1* and *fnr3* deletion), MB231 (triple *fnr* deletion), MBN1 (*nifA* deletion), MBN2 (*nifA* deletion in the *fnr1, fnr3* deletion background) and MBN3 (*nifA* deletion in the triple *fnr* deletion background) harbouring the plasmid pEMS140 (*nifB::lacZ*) were assayed for β-Galactosidase activity under nitrogen deficient media and 4.0% (red bars) or 20.8% (blue bars) of oxygen as described in Methods.
