# Supplementary material for: Enhanced oxygen consumption in Herbaspirillum seropedicae fnr mutants leads to increased NifA mediated transcriptional activation
Source: BMC Microbiol. 2015 May 7;15:95. doi: 10.1186/s12866-015-0432-6 (PMC4422417; doi:10.1186/s12866-015-0432-6)
Supplement: Additional file 3: — Construction and validation of nifA- 3xFlag strains in different H. seropedicae backgrounds. (A) Schematic representation of C-terminally 3xFlag tagged construct and primers (dotted arrows) designed to validate the mutants. Drawings are not to scale. (B) Genotypic validation of strains MBN4 (nifA-3xFlag), MBN5 (nifA-3xFlag in the double fnr1 and fnr3 deletion background) and MBN6 (nifA-3xFlag in the triple fnr mutant background). PCR was performed by using primers flanking the C-terminal region around the insertion of the 3xFlag (as indicated in A). Lanes: 1, 1 Kb ladder Fermentas; 2, no template control; 3, SmR1; 4, suicide vector (pK18nifAFlag); 5, 6 and 7, intermediate strains for SmR1, MB13 and MB231 backgrounds; 8,9 and 10, final 3xFlag tagged strains for SmR1, MB13 and MB231 backgrounds. The fnr genotypes on different nifA-3XFlag backgrounds were verified as showed on Additional file 1. On the left are indicated the length in base pairs (bp) of the DNA ladder. [file 12866_2015_432_MOESM3_ESM.pdf]

Additional file 3.

A

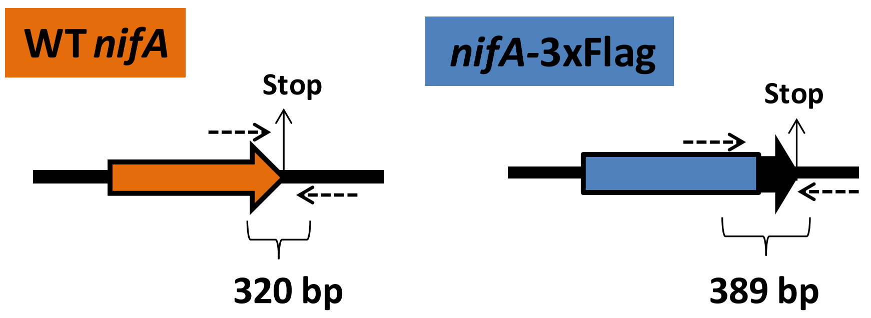

B

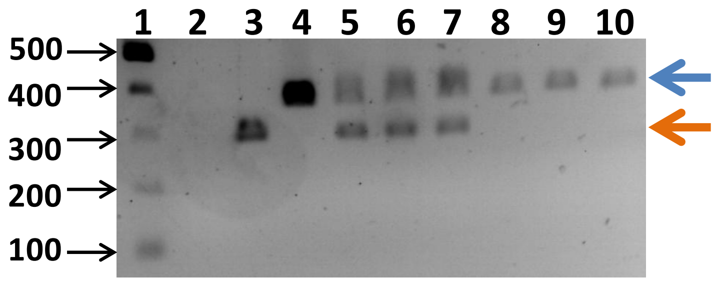

Additional file 3. Construction and validation of *nifA*-3xFlag strains in different *H. seropedicae* backgrounds. (A) Schematic representation of C-terminally 3xFlag tagged construct and primers (dotted arrows) designed to validate the mutants. Drawings are not to scale. (B) Genotypic validation of strains MBN4 (*nifA*-3xFlag), MBN5 (*nifA*-3xFlag in the double *fnr1* and *fnr3* deletion background) and MBN6 (*nifA*-3xFlag in the triple *fnr* mutant background). PCR was performed by using primers flanking the C-terminal region around the insertion of the 3xFlag (as indicated in *A*). Lanes: 1, 1 Kb ladder Fermentas; 2, no template control; 3, SmR1; 4, suicide vector (pK18nifAFlag); 5, 6 and 7, intermediate strains for SmR1, MB13 and MB231 backgrounds; 8,9 and 10, final 3xFlag tagged strains for SmR1, MB13 and MB231 backgrounds. The *fnr* genotypes on different *nifA*-3XFlag backgrounds were verified as showed on Additional file 1. On the left are indicated the length in base pairs (bp) of the DNA ladder.
