# Supplementary material for: Enhanced oxygen consumption in Herbaspirillum seropedicae fnr mutants leads to increased NifA mediated transcriptional activation
Source: BMC Microbiol. 2015 May 7;15:95. doi: 10.1186/s12866-015-0432-6 (PMC4422417; doi:10.1186/s12866-015-0432-6)
Supplement: Additional file 4: — Deletion of fnr genes influences the diazotrophic growth profile. Both H. seropedicae wild type strain (SmR1) (black squares) and the triple fnr mutant strain (MB231) (red triangles) were incubated statically at 30°C in NFbHP-Malate minimal media without addition of nitrogen source. [file 12866_2015_432_MOESM4_ESM.pdf]

Additional file 4.

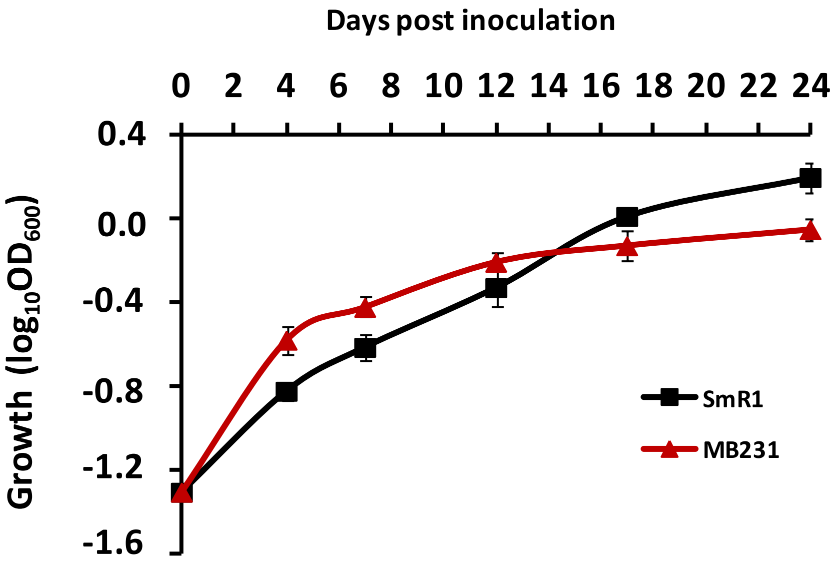

Additional file 4. Deletion of *fnr* genes influences the diazotrophic growth profile. Both *H. seropedicae* wild type strain (SmR1) (black squares) and the triple *fnr* mutant strain (MB231) (red triangles) were incubated statically at 30°C in NFbHP-Malate minimal media without addition of nitrogen source.
