# Supplementary material for: Enhanced oxygen consumption in Herbaspirillum seropedicae fnr mutants leads to increased NifA mediated transcriptional activation
Source: BMC Microbiol. 2015 May 7;15:95. doi: 10.1186/s12866-015-0432-6 (PMC4422417; doi:10.1186/s12866-015-0432-6)
Supplement: Additional file 5: — Construction and validation of fnr1- 3xFlag strain from H. seropedicae. (A) Schematic representation of C-terminally 3xFlag tagged construct and primers (dotted arrows) designed to validate the mutant. Drawings are not to scale. (B) Genotypic validation of the strain MBF1 (fnr1- 3xFlag). PCR was performed by using primers flanking the C-terminal region around the insertion of the 3xFlag (as indicated in A). Lanes: 1, 1 Kb ladder Fermentas; 2, SmR1; 3, suicide vector (pJQfnr1Flag); 4, intermediate strain and 5 final fnr1-3xFlag tagged strain (MBF1). On the left are indicated the length in base pairs (bp) of the DNA ladder. [file 12866_2015_432_MOESM5_ESM.pdf]

Additional file 5.

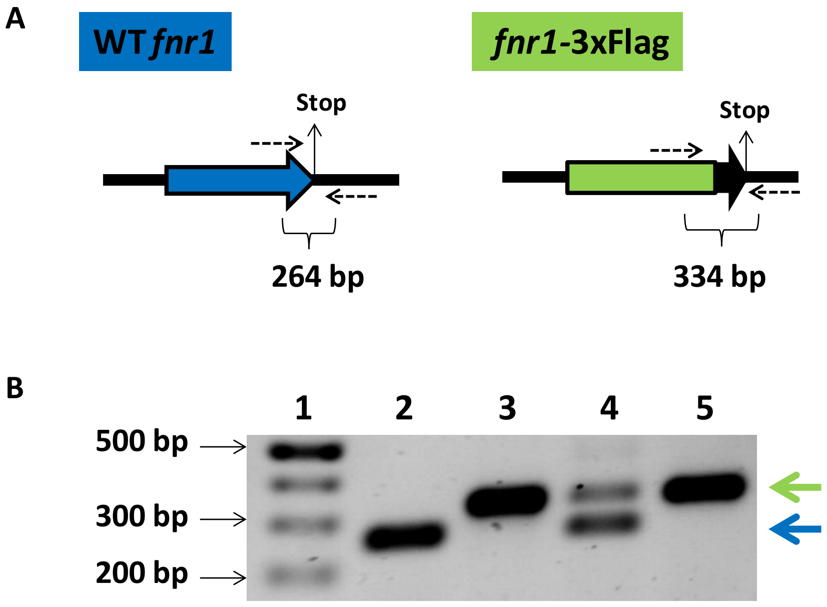

Additional file 5. Construction and validation of *fnr1*-3xFlag strain from *H. seropedicae*. (A) Schematic representation of C-terminally 3xFlag tagged construct and primers (dotted arrows) designed to validate the mutant. Drawings are not to scale. (B) Genotypic validation of the strain MBF1 (*fnr1*-3xFlag). PCR was performed by using primers flanking the C-terminal region around the insertion of the 3xFlag (as indicated in A). Lanes: 1, 1 Kb ladder Fermentas; 2, SmR1; 3, suicide vector (pJQfnr1Flag); 4, intermediate strain and 5 final *fnr1*-3xFlag tagged strain (MBF1). On the left are indicated the length in base pairs (bp) of the DNA ladder.
