# Supplementary material for: Enhanced oxygen consumption in Herbaspirillum seropedicae fnr mutants leads to increased NifA mediated transcriptional activation
Source: BMC Microbiol. 2015 May 7;15:95. doi: 10.1186/s12866-015-0432-6 (PMC4422417; doi:10.1186/s12866-015-0432-6)
Supplement: Additional file 6: — Primers used in this study. [file 12866_2015_432_MOESM6_ESM.pdf]

Additional file 6. Primers used in this study.

Primer	RS[a]	Sequence (5'-->3')[b]	Aplication
CCFlagF	BamHI	*ATATTA***GGATCC**ATGGCCACTATTCTCGAC	Amplification of 1629 bp corresponding to the coding region of the *nifA* gene
CCFlagR	KpnI	*ATATTA***GGTACC**GAACTTCTTGACCTCGAT	
DCFlagF	HindIII	*ATATTA***AAGCTT**AACTCATCTGCGAGCGTG	Amplification of 647 bp corresponding to the downstream region of the *nifA* gene
DCFlagR	XmaI	*ATATTA***CCCGGG**GTTGCACTGGATGTT	
DBamCFlagR	BamHI	*ATATTA***GGATCC**GTTGCACTGGATGTT	Introduce BamHI site at 3' of 3xFlag construct
NifACFlagF	None	ATGGCCAAGGACAAGCCA	Genotypic validation of *nifA*-3XFlag strains
NifACFlagR	None	AATGCGAGGACGTGCCAG	
Fnr1CodF#	BamHI	*TATATA***GGATCC**TTGCCACATGTCCGCCGG	Amplification of 885 bp covering to the coding region of the *fnr1* gene
Fnr1CodR#	Xho I	*ATATAT***CTCGAG**GCTGGTGCTGCGGGTCG	
Fnr1DownF#	HindIII	*TATATA***AAGCTT**GGGCTGGCCTCATCG	Amplification of 1002 bp corresponding to the downstream region of the *fnr1* gene
Fnr1DownR#	XmaI	*ATATAT***CCCGGG**CGCTGATCGGACCCG	
1FlagF	None	ATTGAAAGCATCAGCCGGCTGATC	Genotypic validation of *fnr1*-3XFlag strains
2FlagR	None	CAGACCTTGCCCAGGGAACGT	
NifAdelF	None	ATGGCCACTATTCTCGACGAC	Genotypic validation of *nifA* deletion strains
NifAdelR	None	CTGGTTCTCCACACGGAAT	
nifH-RT-F	None	AAGTCCACCACCTCGCAAAA	RT-PCR
nifH-RT-R	None	TTCCAGCTCCAGATCCTCCA	
16S-RT-F[c]	None	TGTCAGGGAAGAAACGGTTTTG	RT-PCR
16S-RT-R[c]	None	AGTTAGCCGGTGCTTATTCTTCA	

All primers were designed for this study, except when stated.

[a] Restriction site

[b] When present, the restriction sites and non-homologous regions are in bold and italicized, respectively.

[C] Michelle Zibetti Tadra Sfeir, personal communication.
